# Key Markers Involved in the Anticolon Cancer Response of CD8+ T Cells through the Regulation of Cholesterol Metabolism

**DOI:** 10.1155/2021/9398661

**Published:** 2021-11-23

**Authors:** Liang Dong, Xi Yang, Yangyanqiu Wang, Yin Jin, Qing Zhou, Gong Chen, Shuwen Han

**Affiliations:** ^1^Department of Gastroenterology, Huzhou Central Hospital, Affiliated Central Hospital Huzhou University, Sanhuan North Road No. 1558, Wuxing District, Huzhou 313000, Zhejiang, China; ^2^Department of Intervention and Radiotherapy, Huzhou Central Hospital, Affiliated Central Hospital Huzhou University, Huzhou 313000, Zhejiang, China; ^3^Graduate School of Medical College of Zhejiang University, Kaixuan Road No. 268, Jianggan District, Hangzhou 310029, Zhejiang, China; ^4^Department of Laboratory Medicine, Huzhou Central Hospital, Affiliated Central Hospital Huzhou University, Huzhou 313000, Zhejiang, China; ^5^Department of Nursing, Huzhou Central Hospital, Affiliated Central Hospital Huzhou University, Sanhuan North Road No.1558, Wuxing District, Huzhou 313000, Zhejiang, China; ^6^Undergraduate School of Clinic Medicine, Huzhou University, Huzhou 313000, Zhejiang, China; ^7^Department of Oncology, Huzhou Central Hospital, Affiliated Central Hospital Huzhou University, Huzhou 313000, Zhejiang, China

## Abstract

**Background:**

T cell-mediated antitumor immune response is the basis of colorectal cancer (CRC) immunotherapy. Cholesterol plays an important role in T cell signal transduction and function. Apolipoprotein *E* (APOE) plays a major role in cholesterol metabolism.

**Objective:**

To screen and analyze key markers involved in the anticolon cancer response of CD8+ T cells through the regulation of cholesterol metabolism.

**Methods:**

Based on the median cutoff of the expression value of *APOE* according to the data downloaded from The Cancer Genome Atlas and Gene Expression Omnibus database, patients were grouped into low and high expression groups. Differences in clinical factors were assessed, and survival analysis was performed. Differentially expressed genes (DEGs) in the high and low expression groups were screened, followed by the analysis of differences in tumor-infiltrating immune cells and weighted gene coexpression network analysis results. The closely related genes to *APOE* were identified, followed by enrichment analysis, protein–protein interaction (PPI) network analysis, and differential expression analysis. Immunohistochemical staining (IHC) was used to detect the expression of CD8 in CRC tissues.

**Results:**

There were significant differences in prognosis and pathologic_N between the APOE low and high expression groups. A total of 2,349 DEGs between the high and low expression groups were selected. A total of 967 genes were obtained from the blue and brown modules. The probability of distribution of CD8+ T cells differed significantly between the two groups, and 320 closely related DEGs of *APOE* were screened. Genes including the *HLA* gene family, *B2M*, *IRF4*, and *STAT5A* had a higher degree in the PPI network. GEO datasets verified the prognosis and the related DEGs of *APOE*. IHC staining verified the relationship between the distribution of CD8+ T cells and *APOE* expression.

**Conclusion:**

Genes including the *HLA* gene family, *B2M*, *IRF4*, and *STAT5A* might be the key genes involved in the anticolon cancer response of CD8+ T cells through the regulation of cholesterol metabolism.

## 1. Introduction

Colon cancer is a major health problem worldwide because it is the second leading cause of cancer-related deaths [1]. It is reported that by 2030, especially in developed countries, the number of new cases will increase to 2.2 million, and cases of death will increase to 1.1 million [2]. Although surgical resection combined with radiation therapy and chemotherapy is the main treatment for colon cancer, the 5-year survival rate is only approximately 28% [3]. Excessive dietary fat is associated with the induction/exacerbation of various diseases, including colon cancer [4]. In addition, numerous studies have illustrated that a “Western” style diet with high-fat content is an important factor in the increased incidence of colon cancer [5–7]. A high-fat diet is also widely involved in various pathological conditions, such as obesity and metabolic diseases, which may promote the development of colon cancer [8]. Hyperlipidemia is often divided into four types: hypercholesterolemia, hypertriglyceridemia, mixed hyperlipidemia, and low high-density lipoprotein cholesterol. A previous study indicated that hyperlipidemia is a risk factor for colon cancer [9].

T cell-mediated antitumor immune response is the basis of tumor immunotherapy, which is associated with a favorable prognosis [10]. However, some tumors evolve to acquire immunosuppressive properties and escape the attack by T cells through various mechanisms in the tumor microenvironment [11]. Thus, reactivating the cytotoxicity of T cells is of great clinical interest in cancer immunotherapy. Cholesterol plays an important role in T cell signal transduction and function. For instance, Yang et al. found that the antitumor response of mouse CD8+ T cells can be enhanced by regulating cholesterol metabolism [12]. Ma et al. showed that high cholesterol can facilitate T cell immune checkpoint expression, which makes it easier for T cells to lose their antitumor function [13]. However, these studies only focused on T cell gene changes and endoplasmic reticulum stress and did not examine the impact of tumor cells.

**Figure 1:**
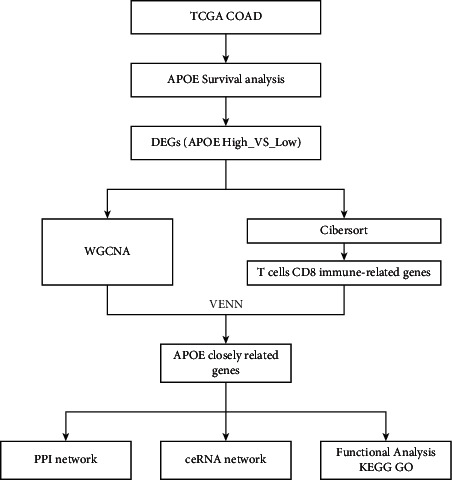
The workflow of this study.

It has been reported that high cholesterol levels can promote the proliferation of stem cells, thereby increasing the growth rate of intestinal tumors by 100-fold [14]. ROR*α*/hdac [15], CD36 [16], ACAT1 [17], and so on are involved in the regulation of the antitumour response of CD8+ T cells by modulating cholesterol metabolism. However, the key markers involved are still unclear. Thus, this study aimed to screen and analyze the key markers involved in the anticolon cancer response of CD8+ T cells through the regulation of cholesterol metabolism. The workflow of this study is shown in Figure 1.

## 2. Materials and Methods

### 2.1. Data Sources and Data Preprocessing

Gene expression RNAseq data [log_2_(fpkm+1)] and clinical data of colon adenocarcinoma were obtained from The Cancer Genome Atlas database [18] (https://xenabrowser.net/). After sequencing data and clinical information matching, a total of 389 tumor samples were obtained (data version: 07-19-2019). RNA-Seq was annotated based on the annotation file of the Gencode database [19] (V23, https://www.gencodegenes.org/). In the expression profile analysis of Ensembl_ID, the mapping probe was used to calculate the gene expression value (obtained from the annotation files of the chip platform and microarray dataset) to Symbol_ID. The average value was taken as the level of Ensembl_ID expression when multiple probes matched one Symbol_ID. Genes with “protein_coding” annotations were then extracted as mRNAs.

### 2.2. APOE Expression-Related Clinical and Survival Analysis

Apolipoprotein *E* (APOE) plays a major role in cholesterol metabolism [20]. Thus, in this study, to screen the key markers involved in the anticolon cancer response of CD8+ T cells via cholesterol metabolism regulation, the expression level of APOE was calculated using the Survminer package of *R* (version: 0.4.8, https://CRAN.Rproject.org/package=survminer), followed by the median cutoff of the expression value. Then, based on the median cutoff of the expression value, patients were grouped into low and high expression groups. In addition, the Kaplan–Meier survival curve analysis and log-rank test were performed to compare the prognosis between the two groups based on the survival information of the samples. Clinical factors (age, sex, TNM stage, and pathologic_stage) were compared between the low and high expression groups using the chi-squared test; differences were considered significant at a threshold of *P* < 0.05.

### 2.3. Differential Expression Analysis

The typical Bayesian method in the limma package [21] (version 3.40.6) was used to analyze differentially expressed genes (DEGs) between high and low expression groups. The Benjamini and Hochberg method was used to adjust the *P* values. Adjusted *P* value < 0.05 and |logFC| > 0.263 were used as the cutoff criteria for screening DEGs. Finally, the ggscatter function of the ggpubr package [22] in *R* (version: 0.2.2) was used to draw a volcano plot.

### 2.4. Screening of APOE Expression-Related Modules and Genes

The weighted gene coexpression network analysis (WGCNA) algorithm was used to screen the modules and genes related to APOE expression based on the expression values of DEGs. The network construction and module screening procedures included dataset consistency analysis and gene coexpression correlation matrix, adjacent function, phase difference between nodes, and correlation analyses between network modules and diseases. A heat map was used to visualize the correlations of each module.

### 2.5. Screening of Immune-Related Genes

The CIBERSORT algorithm [23], a useful method for obtaining high-throughput characteristics of 22 cell types in complex tissues, was used to analyze the abundance of tumor-infiltrating immune cells with the parameters of perm = 100 and QN = *F*. Subsequently, the abundance of all types of immune cells was tested in the high and low expression groups. Then, the Pearson correlation coefficient between DEGs and CD8+ T cells was calculated, and immune-related genes were obtained with a cutoff of *P* < 0.05 and |r| > 0.3. The module genes obtained from WGCNA were intersected with these immune-related genes, and the overlapping genes were redefined as closely related genes of *APOE*.

### 2.6. Enrichment Analysis

Based on the closely related genes of *APOE*, the cluster Profiler package [24] in *R* was used to perform Gene Ontology (GO) [25] and Kyoto Encyclopedia of Genes and Genomes (KEGG) [26] pathway enrichment analyses with a cutoff value of *P* < 0.05 and count ≥2.

### 2.7. Protein-Protein Interaction (PPI) Network Analysis

The STRING database [27] was used to evaluate the PPIs encoded by closely related genes of *APOE*. The PPI score was set at 0.9. Subsequently, the PPI network of T cell-related genes was analyzed using Cytoscape software [28].

### 2.8. Analysis of DEGs Related to APOE and CD8+ T Cells

*R* software ggplot2 (version: 3.2.1, https://CRAN.R-project.org/package=ggplot2) and GGpubr ggpubr (version 0.2.2, *t*-test provided by https://CRAN.R-project.org/package=ggpubr) were used to analyze the differences of genes between the two groups. A box diagram was drawn for display with a threshold of *P* < 0.05.

### 2.9. Datasets Validation

Two GEO datasets, GSE71187 and GSE39582, with prognostic information were selected from the GEO database to validate the result of APOE expression-related survival analysis. The series_matrix.txt data containing 99 tumors and 12 para-cancers was obtained after pretreatment of GSE71187. Furthermore, the series_matrix.txt containing 566 tumors and 19 para-cancers was obtained after pretreatment of GSE39582. The expression of APOE in the two datasets was extracted. *R* Software Survminer (version 0.4.8) package calculated APOE expression to obtain optimal cutoff. The expression value > optimal cutoff was regarded as high expression, and the expression value ≤ optimal cutoff was regarded as low expression. Then, the prognosis of patients in the two groups was obtained. Combined with the survival information of the samples, the k-M curve was plotted, and log-rank was used to test its significance.

Patients in GSE71187 and GSE39582 were divided into two groups according to the median APOE expression. The expressions of transcription 5A (STAT5A), histocompatibility leukocyte antigen (HLA) gene family (HLA-E, HLA-C, HLA-B, etc.), beta-2 microglobulin (B2M), and interferon regulatory Factor 4 (IRF4) were tested in high and low APOE expression groups. *R* software GGplot2 (version 3.2.1, https://CRAN.R-project.org/package=ggplot2) and GGpubr ggpubr (version 0.2.2, the *t*-test provided by https://CRAN.R-project.org/package=ggpubr) were used to analyze the differences of genes between the two groups. A box diagram was drawn for display with a threshold of *P* < 0.05.

### 2.10. CRC Specimens and IHC Staining

The study protocol was approved by the Human Ethics Committee of Huzhou Central Hospital (NO: 20210207). Forty CRC patients were recruited, and the basic information was presented in Supplementary Table 2. The expression of APOE in blood was detected by using immunoturbidimetry. APOE higher than 53.0 mg/L was included in the APOE-high group, and APOE lower than 29.0 mg/L was included in the APOE-low group. Further detection of CD8 expression was conducted by using immunohistochemistry. Immunohistochemistry test kit, antigen repair solution, and CD8 antibody were all purchased from Beijing Zhongshan Jinqiao Biotechnology Co., Ltd. The tissue sections were scanned at low magnification (X100) to select the areas with high CD8 positive cell density. Then, the positive rate of CD8 cells in five fields was determined at high magnification (X200) to calculate the average value. The positive rate of CD8 cells was expressed as mean ± standard deviation. Using SPSS 13.0 statistical software, the Pearson *χ*2 test was performed.

## 3. Results

### 3.1. Comparison of Clinical Factors and Analysis of DEGs between the High and Low Expression Groups

**Figure 2:**
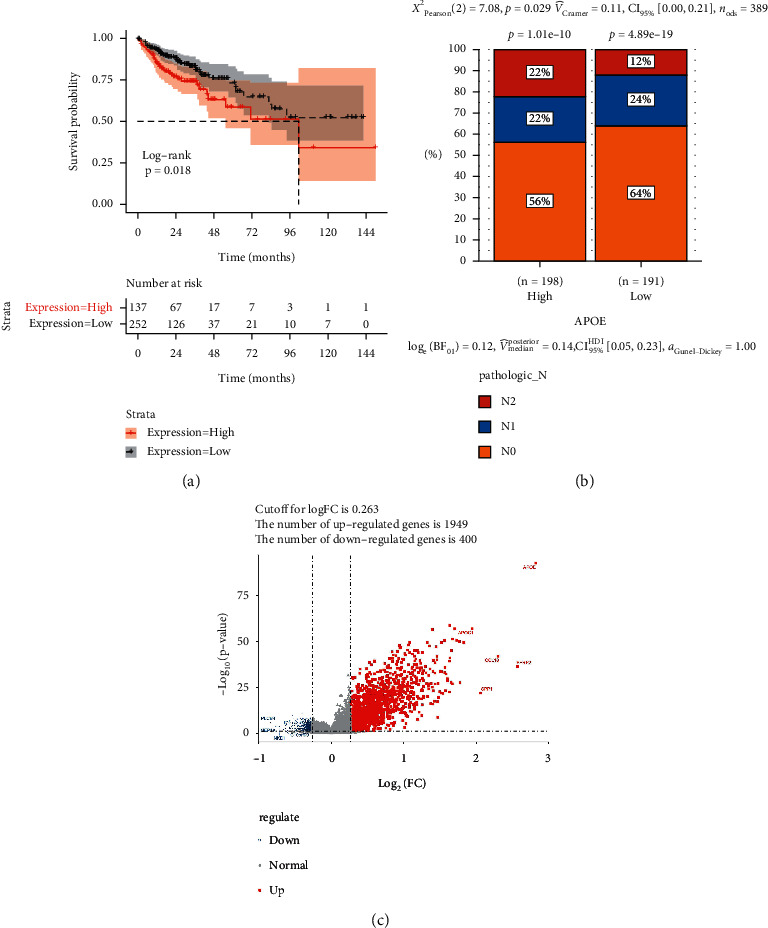
Comparison of clinical factors, survival analysis, analysis of DEGs between the high and low expression groups. (a) Survival analysis between low and high expression groups. (b) Comparison of clinical factors (age, sex, TNM stage, pathologic_stage) between the low and high expression groups. (c) DEGs between low and high expression groups; the red nodes represent upregulated DEGs, and the blue nodes represent downregulated DEGs.

The Kaplan–Meier survival curve analysis and log-rank test were performed to compare the prognosis between the two groups based on the survival information of the samples. The results showed that there were significant differences in prognosis between the low and high expression groups (*P* = 0.018) (Figure 2(a)). In addition, a significant difference in pathologic N was found between the two groups (*P* = 0.029) (Figure 2(b)). In total, 2,349 DEGs, of which 1,949 and 400 were upregulated and downregulated, respectively, between the high and low expression groups were selected (Figure 2(c)).

### 3.2. Disease-Related Modules and Genes

**Figure 3:**
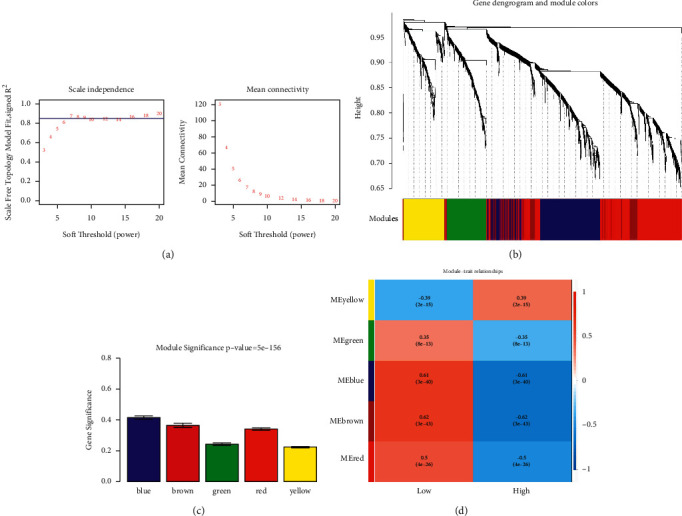
Disease-related modules and genes. (a) Determination of the soft threshold for the adjacency matrix. The horizontal axis represents the soft threshold power, and the vertical axis represents the square of the correlation coefficient between log_2_k and log_2_p (k). The blue line indicates a correlation coefficient of 0.85 and a corresponding soft threshold power of 8. (b) Gene dendrogram derived from hierarchical clustering. Different modules are indicated by the colors below the dendrogram. (c) Gene significance (GS) of five modules. (d) Module–disease relationships. The modules are represented by different colors along the horizontal axis. Correlation coefficients are indicated as numbers in corresponding positions, and *p* values are shown in brackets along the coefficients. Red indicates higher correlation coefficients.

A total of 2,349 DEGs were used for WGCNA analysis. The soft threshold power for matrix transformation was determined to be 8, with the square of the correlation coefficient between log2k and log2p (*k*) being 0.85 (Figure 3(a)). The minimum number of genes was set to 30 for each module, and the pruning height was cutHeight = 0.2. A total of five different disease-related modules were screened (Figure 3(b)), including blue, brown, green, red, and yellow modules. To ensure the reliability of the screening results of key network modules, the disease-related key network modules were analyzed again by calculating the absolute value of gene significance (GS) within the module, and the results showed that the blue and brown modules had a higher GS (Figure 3(c)) and were significantly correlated with the disease (Figure D). Finally, a total of 967 genes were obtained from the blue and brown modules.

### 3.3. CD8+ T Cell-Related DEGs

**Figure 4:**
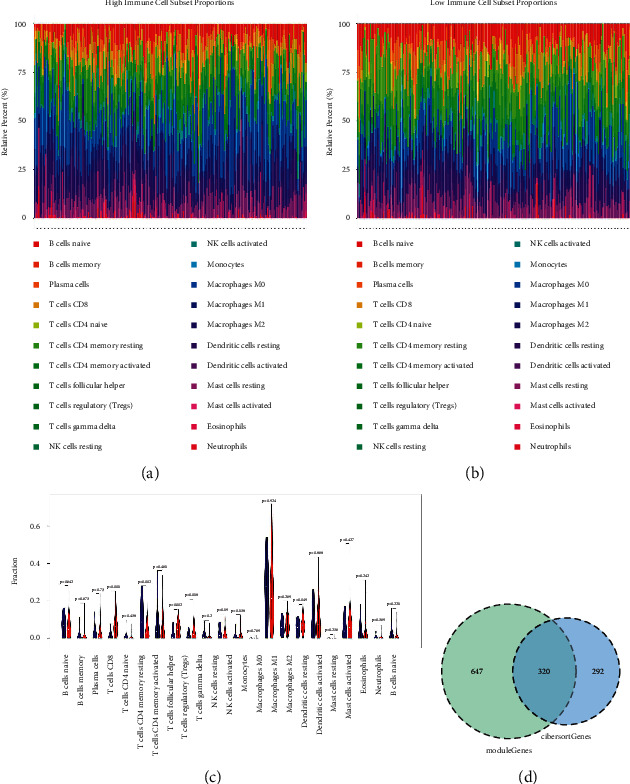
Screening of CD8+ T cell-related DEGs. (a) The 22 tumor-immune cell proportions of high expression groups. (b) The 22 tumor-immune cell proportions of low expression groups. (c) The violin plot of different tumor-immune cells between high and low expression groups; blue represents the high expression group, and red represents the control group. (d) The Venn diagram of closely related DEGs of *APOE*. WGCNA: weighted gene coexpression network analysis; APOE: apolipoprotein E.

Immune infiltration in the high and low expression groups was analyzed based on the CIBERSORT algorithm. As illustrated in Figures 4(a) and 4(b), 22 tumor-immune cell proportions in the high and low expression groups were analyzed. To observe the probability of the distribution of different tumor-immune cells between the two groups, a violin plot was drawn. As shown in Figure 4(c), the probability of the distribution of CD8+ T cells was the most significant difference between the two groups (*P* < 0.01). The correlation coefficient between DEGs and CD8+ T cell-related genes was then calculated, and a total of 612 CD8+ T cell-related DEGs were identified. In addition, the module genes obtained from WGCNA intersected with these CD8+ T cell-related genes, and a total of 320 closely related DEGs of *APOE* was obtained (Figure 4(d), Supplementary Table 1).

### 3.4. Enrichment Analysis and PPI Network Analysis of Closely Related DEGs of APOE

**Figure 5:**
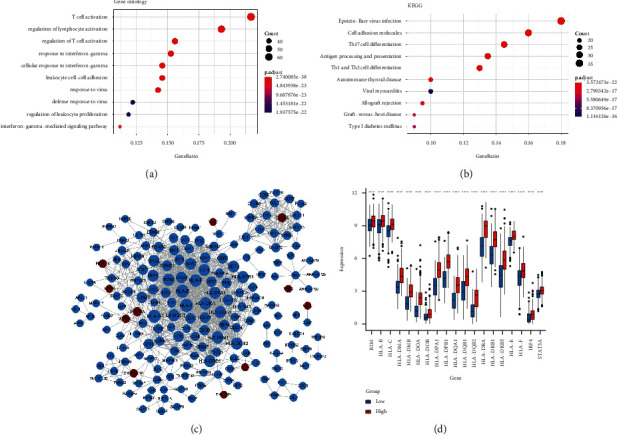
Enrichment analysis, PPI network analysis, and DEGs related to APOE and CD8+ T cells. (a) GO-BP. (b) KEGG pathway. The size of the ball represents the number of genes enriched in each term. The color of the ball represents the value of the *P* value. (c) PPI network; blue nodes represent the genes in the blue module, and brown nodes represent genes in the brown module. (d) Analyses of DEGs related to APOE and CD8+ T cells.

Enrichment analysis showed that the closely related upregulated DEGs were mainly involved in 544 GO-biological processes (BPs; e.g., T cell activation; regulation of lymphocyte activation; and regulation of T cell activation) and 54 KEGG pathways (e.g., hsa05169: Epstein–Barr virus (EBV) infection and hsa04514: cell adhesion molecules) (Figures 5(a) and 5(b)), whereas the closely related downregulated DEGs were not involved in the GO-BP and KEGG pathways. PPI network analysis of 320 closely related DEGs of *APOE* revealed 191 nodes and 1,153 interaction pairs (Figure 5(c)). In this PPI network, genes including the histocompatibility leukocyte antigen (HLA) gene family (HLA-E, HLA-C, HLA-B, etc.), beta-2-microglobulin (*B2M*), interferon regulatory factor 4 (*IRF4*), and STAT5A had a higher degree, which could lead to their classification as key genes (Table 1).

### 3.5. Analysis of DEGs Related to APOE and CD8+ T Cells

As shown in Figure 5(d), histocompatibility leukocyte antigen (HLA) gene family (HLA-E, HLA-C, HLA-B, etc.), beta-2-microglobulin (B2M), interferon regulatory factor 4 (IRF4), and STAT5A were significantly differentially expressed in high and low APOE groups (*P* < 0.001).

### 3.6. Validation of APOE-related survival analysis and DEGs related to APOE and CD8+ T cells

**Figure 6:**
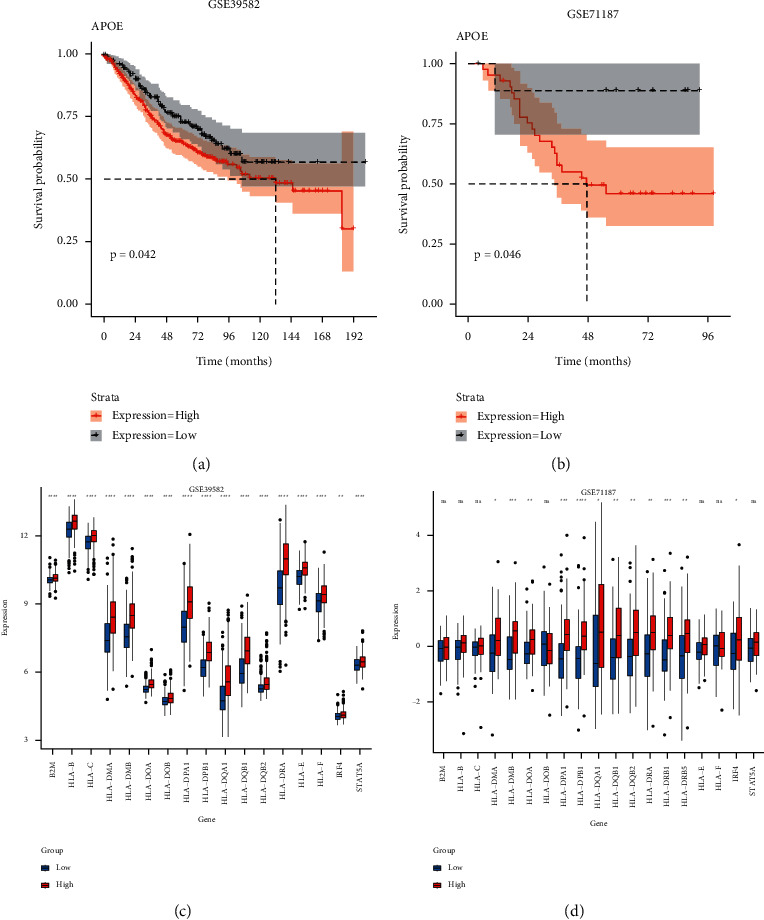
Validation of survival analysis and DEGs related to APOE and CD8+ T cells. A-B: survival analysis between APOE low and high expression groups in GSE39582 and GSE71187. C-D: analysis of DEGs related to APOE and CD8+ T cells in GSE39582 and GSE71187.

As shown in the K-M curve in Figures 6(a) and 6(b), the survival analysis results of GSE3958 and GSE71187 were verified that the high expression group had a poor prognosis (*P* < 0.05). Moreover, as shown in the box diagram in Figures 6(c) and 6(d), most of the concerned genes in the GSE39582 and GSE71187 datasets were highly expressed (*P* < 0.05). The results were consistent with the analysis results of the TCGA database.

### 3.7. The Infiltration of CD8+T Cells Correlated with APOE in Tissue Specimen Sections

**Figure 7:**
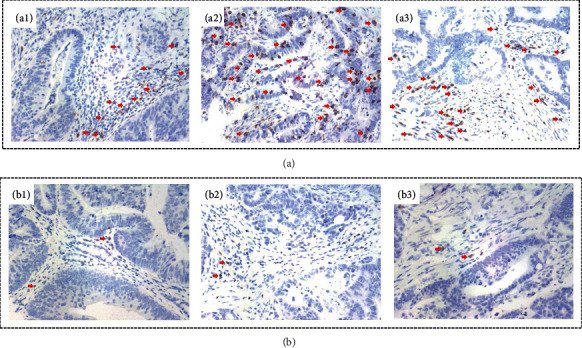
IHC staining of CD8. (a). IHC staining for CD8 in the high-APOE expression group. (b). IHC staining for CD8 in the low-APOE expression group. The magnification was X200. The red arrows represented positive CD8.

The basic information of recruited CRC patients was present in Supplementary Table 2. IHC staining detection of CD8 proteins showed that CD8 was upregulated expression in the APOE-high expression group (Figure 7(a)), while CD8 was downregulated expression in the APOE-low expression group (Figure 7(b)) (*P* = 0.001). The statistical results are presented in Table 2.

## 4. Discussion

In this study, there were significant differences in prognosis and pathologic_N between the low and high expression groups. A total of 2,349 DEGs between the high and low expression groups were selected. In addition, the probability of CD8+ T cell distribution was the most significant difference between the two groups. A total of 320 closely related DEGs of *APOE* were identified. Genes including the *HLA* gene family, *B2M*, and *IRF4* had a higher degree in the PPI network, and *STAT5A* was regulated by numerous miRNAs in the ceRNA network.

Cholesterol has been reported to be associated with colon cancer. Eggs low in fat contain large amounts of cholesterol, and egg consumption has been associated with an increased risk of colon cancer [29]. In addition, Jacobs et al. showed that high cholesterol levels seem to increase the risk of colon cancer [30]. In line with our data, in this study, the survival analysis results showed that the high cholesterol group was related to poor survival, which suggested that high cholesterol was a risk factor for colon cancer. In addition, a significant difference in pathologic_N was found between the low and high expression groups, and the proportion of pathologic_N3 in the high expression group was higher than that in the low expression group, which further indicated that high cholesterol was a risk factor for colon cancer.

CD8+ T cells play an extremely crucial role in antitumor immunity; however, tumor-infiltrating T cells often lose their effector functions. Ma et al. reported that cholesterol induced CD8+ T cell exhaustion by modulating endoplasmic reticulum stress pathways (IRE1/XBP1) in the tumor microenvironment [13]. What is more, CD8+ T cells were polarized into IL-9-secreting (Tc9) cells to exert antitumor responses, while cholesterol or its derivatives inhibited IL-9 expression by activating liver *X* receptors (LXRs) and leading to LXR SUMOylation and reduced p65 binding to IL-9 promoter [31]. The characteristics of exhausted CD8+ T cells were attenuating antitumor responses and decreasing infiltrating density. Therefore, high cholesterol decreased the infiltration of CD8+ T cells. In this study, we also analyzed the key markers involved in the anticolon cancer response of CD8+ T cells through cholesterol metabolism regulation using bioinformatics analysis. The results showed that genes including the *HLA* gene family, *B2M*, and *IRF4* had a higher degree in the PPI network, and *STAT5A* was regulated by numerous miRNAs in the ceRNA network. The *HLA* gene family plays a major role in regulating immune responses in cancers. For instance, Tsai et al. revealed that *HLA-DQA1* and *HLA-DQB1* may participate in the development of oral cancer [32]. Bianchini et al. showed that the *HLA-E* gene strongly supports a potential tumor-evading immune surveillance strategy in colon cancer tissues [33]. Moreover, Benevolo et al. found that the *HLA-A*, *HLA-B*, and *HLA-C* expression in colon cancer is associated with prognosis [34]. Blum et al. suggested that, in patients with stage II colon cancer, the Map7/B2M expression ratio is a prognostic factor for survival [35]. *IRF4* is considered an oncogene in lymphoid malignancy and multiple myeloma [36], and Zhang et al. showed that overexpression of circPIP5K1A attenuated the expression of *IRF4* in the progression of colon cancer development [37]. As a transcription factor, the activation or phosphorylation of STAT is associated with many cancers. Slattery et al. showed that STAT5A was associated with colon cancer progression [38]. In addition, nucleotide analysis showed that the upregulated closely related genes were involved in EBV infection and cell adhesion molecule KEGG pathways. EBV is associated with several malignant tumors. Guan et al. reported a positive association between EBV infection and colon cancer [39]. It has been reported that a variety of cell adhesion molecules are involved in cell–cell and cell–matrix interactions in colon cancer, and some cell–cell and cell–matrix interactions determine colon cancer behavior [40]. Based on these results, we speculate that genes including the *HLA* gene family, *B2M*, *IRF4*, and *STAT5A* that may be involved in EBV infection and cell adhesion molecule pathways play important roles in the anticolon cancer response of CD8+ T cells by regulating cholesterol metabolism.

However, there are some limitations to this study. We need to conduct related experiments such as cell biology experiments and animal studies to validate the multiple candidate targets and signaling pathways obtained in this bioinformatics analysis.

## 5. Conclusion

In summary, genes including the *HLA* gene family, *B2M*, *IRF4*, and *STAT5A* might be the key genes involved in the anticolon cancer response of CD8+ T cells via regulation of cholesterol metabolism. These findings can guide clinical decision-making for colon cancer treatment as well as future research on colon cancer.

## Figures and Tables

**Table 1:** The degree of top 20 nodes in protein-protein interaction (PPI) network.

Gene	Degree
HLA-E	52
HLA-C	51
HLA-B	51
HLA-F	47
HLA-DRB1	45
HLA-DRA	45
B2M	44
HLA-DQA1	42
HLA-DQB1	42
HLA-DRB5	42
HLA-DQB2	42
HLA-DPA1	42
HLA-DPB1	42
IRF4	40
IRF1	39
OASL	38
OAS2	38
OAS3	37
GBP2	37
TRIM21	35

**Table 2:** Immunohistochemical evaluation.

CRC	High-APOE	Low-APOE	Total	*X* ^2^	*P* value
CD8 ++/+++	18	8	26	—	—
CD8−/+	2	12	14	—	—
Total	20	20	40	10.989	0.001

Criterion: —: 0–10%; +: 11–30%; ++: 31–70%; +++: 70–100%.

## Data Availability

Raw data were obtained from TCGA repositories and https://xenabrowser.net/datapages/. Validation data were obtained from GEO repositories (GSE3958 and GSE71187) (https://www.ncbi.nlm.nih.gov/geo). Analyzed data are available within supplementary information or from the authors upon reasonable request.

## Abbreviation

KEGG: Kyoto Encyclopedia of Genes and Genomes
GO: Gene Ontology
GEO: Gene Expression Omnibus
PPI: Protein–protein interaction
BP: Biological process
ceRNA: Competing endogenous RNA
WGCNA: Weighted gene coexpression network analysis
DEGs: Differentially expressed genes
APOE: Apolipoprotein E
HLA: Histocompatibility leukocyte antigen
STAT5A: Signal transducer and activator of transcription 5A
IRF4: Interferon regulatory factor 4
B2M: Beta-2-microglobulin.

## Additional Points

1. The prognosis of colon cancer differed between the high and low cholesterol groups. 2. The probability of CD8+ T cell distribution differed significantly between the high and low cholesterol groups. 3. Genes including the *HLA* gene family, *B2M*, and *IRF4* might be the key genes involved in the anticolon cancer response of CD8+ T cells by regulating cholesterol metabolism.

## Supplementary Materials

Supplementary Materials Supplementary Table 1. Related DEGs of APOE. Module genes obtained from WGCNA were intersected with CD8+ T cell-related genes, and a total of 320 closely related DEGs of APOE was obtained. Supplementary Table 2. Basic information of 40 CRC patients. Raw data were obtained from TCGA repositories and, for our purposes, from https://xenabrowser.net/datapages/. Analyzed data are available within supplementary information or from the authors upon reasonable request.
