# Supplementary material for: Key Markers Involved in the Anticolon Cancer Response of CD8+ T Cells through the Regulation of Cholesterol Metabolism
Source: J Oncol. 2021 Nov 23;2021:9398661. doi: 10.1155/2021/9398661 (PMC8632400; doi:10.1155/2021/9398661)
Supplement: Supplementary Materials — Supplementary Table 1. Related DEGs of APOE. Module genes obtained from WGCNA were intersected with CD8+ T cell-related genes, and a total of 320 closely related DEGs of APOE was obtained. Supplementary Table 2. Basic information of 40 CRC patients. Raw data were obtained from TCGA repositories and, for our purposes, from https://xenabrowser.net/datapages/. Analyzed data are available within supplementary information or from the authors upon reasonable request. [file 9398661.f1.zip › 9398661.f1/Supplementary Table 1.docx]

APOE

APOC1

C1QA

C1QC

C1QB

SLC15A3

DOK2

HLA-DPB1

LSP1

LILRB4

IL4I1

SLAMF8

HAVCR2

HCST

CCL18

CD300LF

SDS

CD37

ABI3

FCGR1A

TYMP

WAS

RAB42

TNFAIP8L2

CD52

FMNL1

HAPLN3

SIGLEC1

STX11

NFAM1

LIMD2

NCF1

HLA-DPA1

HLA-DQA1

SIGLEC10

MYO1G

GMFG

PARVG

VAMP5

SASH3

TNFRSF4

HLA-DMB

S1PR2

HCLS1

HLA-DOA

CD72

HLA-DRA

ARHGAP30

APBB1IP

FAM78A

ICAM1

ARHGAP9

IL10RA

SELPLG

HLA-DRB1

CD74

CD48

NKG7

LTB

RASAL3

GNGT2

CD7

GIMAP4

ITGAL

IL18BP

HLA-DQB2

FAM26F

CSF1

TNFRSF18

PIK3CD

IL21R

CCL5

SPN

ARHGAP25

CCL4

PLA2G2D

RGS19

EVL

HLA-DMA

TBC1D10C

GZMM

GIMAP1

IL2RB

CCR5

PLEKHF1

GM2A

CORO1A

NFATC1

IL2RA

IFI30

CTSW

CD3E

PSTPIP1

RASSF4

LAG3

SPOCK2

C10orf54

IL16

APOBEC3G

CD247

RAB33A

MAP4K1

HLA-DRB5

IL12RB1

FGD2

KLHDC7B

HLA-DQB1

CD8A

TNFSF13B

GIMAP7

CXCL13

UBE2L6

GZMH

TNFAIP2

CD2

CD40

SLC2A5

APOBEC3H

GZMK

CD3D

CD27

PDCD1

SIRPG

CXCL9

EBI3

PRF1

CXCL10

SLAMF7

LGMN

FLVCR2

ODF3B

SAMHD1

PLCB2

BST2

SECTM1

MAPK11

RASSF5

TIGIT

PPP1R16B

GBP5

SLA2

HLA-E

APOL3

S1PR4

TNFRSF9

IFIT3

APOL4

ZNF683

SEPT1

JAK3

RARRES3

MMP25

AGAP2

CD6

GBP4

RNF166

RAC2

IFI6

UBASH3A

GNLY

IKZF1

CTLA4

ITM2A

ACAP1

OAS2

ICAM3

CEACAM21

MX1

CD70

BATF

STAT2

TRAFD1

C19orf38

CCR7

IDO1

CD96

GBP1

SNAI3

SH2D1A

SIT1

GZMA

TRIM22

USP18

IFITM10

TAP1

LAP3

ZBED2

BATF3

WARS

STAT1

CIITA

P2RY10

PIK3AP1

CD79A

IFIT2

COTL1

TMC8

CDR2L

RSAD2

SYTL3

SLAMF1

CD3G

CXCR6

HLA-C

CST7

ZAP70

IFI16

PSMB9

NR1H3

CD244

PML

UBD

SP140

IGLL5

CX3CL1

ICOS

TRAF3IP3

CXCL11

FASLG

SMAP2

BLVRA

XCL2

ITGB7

CD274

UAP1L1

PLAT

APOL2

EPSTI1

CD82

MAPK12

IRF1

STAT5A

RELT

ITGAE

UCP2

MZB1

DDX58

TRIB2

CMPK2

AIM2

HLA-DOB

BTN3A3

ZMYND15

SEPT6

PARP9

MICB

GPR171

HLA-F

LCK

APOBEC3D

PSME1

IL15RA

ISG20

LAX1

CXCR3

SAMD9L

ETV7

DHX58

HLA-B

C19orf66

CD38

GBP2

ATF5

CCDC109B

SOCS1

JADE2

BATF2

APOL1

RNF19B

OAS3

PARP12

SLAMF6

FAM43A

BTN3A1

FGD3

IL27RA

BTN3A2

IKZF3

XAF1

FBXO6

TRIM21

TRIM69

CBR3

IRF4

TNFRSF1B

TAP2

HPSE

CD8B

FBXL16

IFIH1

TAPBP

PIM2

KLRB1

HERC6

B2M

PSME2

SLC4A11

NLRC5

LAMP3

DDX60

GFI1

PARP14

EPHB6

FOSL1

OSR2

UBA7

RTP4

OASL

PLA2G2A

ASPHD2

LYZ

TRIM7

TNFSF9

GZMB

C2

ZIC2

IRX2

TNNT1

GABRP

GJB5
