# Supplementary material for: Key Markers Involved in the Anticolon Cancer Response of CD8+ T Cells through the Regulation of Cholesterol Metabolism
Source: J Oncol. 2021 Nov 23;2021:9398661. doi: 10.1155/2021/9398661 (PMC8632400; doi:10.1155/2021/9398661)
Supplement: Supplementary Materials — Supplementary Table 1. Related DEGs of APOE. Module genes obtained from WGCNA were intersected with CD8+ T cell-related genes, and a total of 320 closely related DEGs of APOE was obtained. Supplementary Table 2. Basic information of 40 CRC patients. Raw data were obtained from TCGA repositories and, for our purposes, from https://xenabrowser.net/datapages/. Analyzed data are available within supplementary information or from the authors upon reasonable request. [file 9398661.f1.zip › 9398661.f1/Supplementary Table 2.docx]

**Supplementary Table 2** The basic information of recruited CRC patients.

| CRC | High-APOE | Low-APOE | P value |
| --- | --- | --- | --- |
| Number | 20 | 20 | — |
| Male | 10 | 11 | 0.75 |
| Female | 10 | 9 | 0.75 |
| Age | 60.8±5.93 | 61.80±7.95 | 0.66 |
| BMI(Kg/m^2^) | 21.06±1.79 | 20.78±1.78 | 0.62 |
| Known hypertention | 3 | 5 | 0.43 |
| Known diabetes | 2 | 4 | 0.38 |
| Long-term smoking history | 5 | 3 | 0.43 |
| Long-term drinking history | 5 | 4 | 0.71 |

Overall, 40 CRC patients in Huzhou Central Hospital from January 2021 to September 2021 were recruited in the present study. All patients were volunteered for the study. CRC patients with distant organ metastasis, complicating other gut diseases, such as ulcerative colitis and Crohn’s disease, complicating multiple primary tumours, and known primary organ failure were excluded.
